# The English spaced compound word database

**DOI:** 10.3758/s13428-026-03113-x

**Published:** 2026-07-10

**Authors:** Casey M. Riedmann, Barbara J. Juhasz, Simon P. Liversedge, Chuanli Zang

**Affiliations:** 1 Department of Psychology, Wesleyan University, Middletown, CT 06459 USA; 2 School of Psychology, Northumbria University, Newcastle, UK; 3 School of Psychology, Liverpool Hope University, Liverpool, UK

**Keywords:** Compound words, Spaced compound words, Age-of-acquisition, Familiarity, Semantic transparency

## Abstract

Compound words combine existing words to form a new concept. Spaced compound words represent one form of compound words in which the orthographic space is preserved between the two constituent words (e.g., *coffee table*). While recent research has mostly focused on unspaced compound words, spaced compound words offer important insight into the cognitive processes that support lexical retrieval and comprehension. Critically, whether readers process spaced compound words as single lexical units has important implications for how these items are stored and accessed in memory. To support future research, we developed the English Spaced Compound Word Database, wherein we collected familiarity, age-of-acquisition (AoA), and semantic transparency (ST) ratings for 1,162 spaced compound words. We conducted correlational analyses to compare these ratings across words, as well as across previously collected data on compound words’ constituents. These analyses revealed a strong association overall between familiarity ratings and AoA, as well as familiarity and ST. When examining the relationship between ratings and the characteristics of a compound word’s constituents, we found a strong relationship between frequency, AoA, and structural simplicity (i.e., number of characters, syllables, morphemes, and lexical similarity) within all three rating tasks. These findings provide a basis for testing future theoretical models of lexical processing and reading.

Compounding is a word formation process that is highly common in Germanic languages, where two (or more) free lexemes are combined to represent a single concept (see Bybee, 2015, for a discussion). A novel compound word is lexicalized when it becomes known to speakers of a language, producing a new conceptual representation in the mental lexicon. As a compound word is encountered in speech and print, links form between phonological, orthographic, and semantic representations. While compounding is found in many languages, the orthographic rules regarding this process vary.

Compound words are often endocentric, such that the compound’s meaning is determined by a head constituent (Bisetto & Scalise, 2005). In English, this head constituent typically occurs in the second position, such that the first constituent serves as a modifier that narrows the possibilities of what the head can represent. For example, the compound word *raincloud* designates a type of cloud that bears rain, and not the other way around. This modifier-head relationship is a key aspect of how many compound words are created to refer to specific concepts (Benczes, 2005, 2014, 2015; Plag, 2003, Williams, 1981). In English, compound words can be written in three orthographic formats: unspaced (*boyfriend*), spaced (*coffee table*), and hyphenated (*know-how*; see Kuperman & Bertram, 2013). Unspaced compounds (also called “concatenated” or “closed” compounds) typically consist of two lexemes combined to form a single orthographic unit (e.g., *farmhouse, seasick*), although lexicalized tri-constituent compounds do exist (e.g., *nonetheless*; Bertram et al., 2011). Spaced compounds (also called “open” compounds), on the other hand, represent a single concept but are written with a space between their lexemes (e.g., *front door, potato chip*). While spaced compounds can also include more than two lexemes (e.g., *chicken pot pie*), many instances of common two-constituent spaced compounds exist in English and are listed in English dictionaries. Finally, hyphenated compounds link constituents together via a hyphen, with the punctuation serving as an orthographic cue for readers that can help to segment the compound word more efficiently (see Cherng, 2008; Sun & Baayen, 2021).

These distinct orthographic formats may also carry independent syntactic properties (Elsherif & Catling, 2024; Elsherif et al., 2020; Frisson et al., 2008; Kuperman & Bertram, 2013). Unspaced and hyphenated compounds, for instance, may be more likely to be stored and accessed as whole words (e.g., Monsell, 1985). That is, when readers encounter an unspaced compound like *boyfriend*, they can retrieve the lexical entry for *boyfriend* rather than the lexical entries *boy* and *friend* separately, though access to these constituents may influence processing (see next section for further discussion). For spaced compounds, on the other hand, the retrieval process is less clear. By some accounts, the space between words designates independent syntactic units that form larger constituencies (Inhoff et al., 2000; Juhasz, 2005). For example, *coffee table* consists of two nouns that, together, make up a noun phrase. If reading processes are driven by syntax, then one would expect the reader to retrieve the lexical items *coffee* and *table* separately and then compound them to form the spaced compound *coffee table*. In this case, the space between constituents appears to act as a processing cue that signals readers to retrieve the two words independently. However, readers may also store a distinct lexical entry for the compound form, similar to the way idioms are stored and accessed in the lexicon (see Bonin et al., 2018; Sprenger et al., 2006). This means that readers may process both constituents together as a single lexical entry (Kuperman & Bertram, 2013; Cutter et al., 2014; see also Zang, 2019). Despite the space between words, factors including predictability or context may prompt readers to recognize the two words as a compound word that represents a single concept (Schmidtke et al., 2018). As these representations become more prominent within a culture and/or the word pair co-occurrence becomes more frequent and reinforced in memory, the spaced compound may be transformed into an unspaced or hyphenated compound, highlighting a common pattern of diachronic language change (Kuperman & Bertram, 2013). Therefore, one of the primary purposes of the English Spaced Compound Word Database is to provide a list of spaced compound words^1^ and associated metrics as a starting place for processing inquiries.

It is also noteworthy that English contains more examples of spaced compounds relative to other alphabetic languages such as German and Finnish, where multi-constituent novel compounds can be combined into a single orthographic unit (see Inhoff et al., 2000). This level of spelling variability for English compound words can be a source of frustration for native and non-native English speakers alike (see Kuperman & Bertram, 2013, for a discussion). Dictionaries and writing resources such as the American Psychological Association Style Guide (American Psychological Association, 2020) can provide some external validation for how a given compound word should be spaced. However, there is often variability between sources, adding to the potential confusion of how a given compound word should be written.

There now exist multiple sources for selecting concatenated English compound words for use in psycholinguistic research (e.g., Gagné et al., 2019; Juhasz et al., 2015; Kim et al., 2019). These compound word databases have been useful for selecting items for research exploring questions of how compound words are stored and accessed in the mental lexicon (e.g., Elsherif & Catling, 2023, 2024; Elsherif et al., 2020; Juhasz, 2018). However, to our knowledge, a comparable database does not currently exist for English spaced compound words (but see Bonin et al., 2022, for a database of French spaced and hyphenated compounds). Despite their apparent importance for processing accounts, there is greater ambiguity concerning spaced compounds’ status as compound words, as many co-occurring word pairs would not typically be considered compound words. Additionally, the separation of constituents in spaced compounds makes judgments more complicated for traditional measures of compound words. Thus, spaced compounds are also not available from other open-source research tools such as the English Lexicon Project (ELP; Balota et al., 2007). The motivation for the current study, therefore, was to create a database of English two-constituent spaced compound words to aid researchers in selecting stimuli for their studies and performing analyses to explore compound processing. The database also includes newly collected ratings of age of acquisition (AoA), familiarity, and semantic transparency (ST) for these spaced compound words. Below, we briefly discuss past research on compound words and their relationship to the three rated variables in the database.

## English compound word processing

A growing body of psycholinguistic research has focused on the processing of unspaced compound words (e.g., Falkauskas & Kuperman, 2015). One reason for this interest is that the recognition of unspaced compound words can provide information on how morphologically complex words are stored and accessed in a reader’s mental lexicon. Multiple studies, using a variety of paradigms, have supported a functional role of the two constituents in compound word processing. Eye movement studies, for instance, have shown that English compound words with high-frequency constituents are processed faster than compound words with low-frequency constituents (e.g., Andrews et al., 2004; Juhasz, 2012; Juhasz et al., 2003). This suggests that compound words are linked to the representations of their constituents at some level in the mental lexicon. However, research has shown that the frequency of the compound word as a whole unit also plays a role in compound word processing in English (e.g., Andrews et al., 2004; Juhasz, 2008, 2018). This supports the idea that a whole-word representation exists for familiar concatenated compound words. Together, these results suggest that compound words may be recognized through a decomposition route or a whole-word route during reading. Factors such as the length of the compound and its familiarity may impact which route is used for processing a particular compound word (see Hyönä, 2012, for a discussion).

To aid research on recognition of English compound words, several databases have been published in the past decade that include English unspaced compound words along with their properties. Juhasz et al. (2015) published a database of 629 English compound words containing ratings of six psycholinguistic variables (AoA, familiarity, ST, lexeme meaning dominance, imageability, and sensory experience ratings [SER]). The inclusion of these six variables was motivated by previous research showing their effects on recognition of morphologically simple and/or compound words (e.g., Balota et al., 2004). Analyses revealed that all variables except lexeme meaning dominance predicted lexical decision times from the ELP (Balota et al., 2007) when added to a baseline model that accounted for the length and frequency of the whole compound, as well as the frequencies of its constituents. Additionally, four variables impacted naming times (AoA, familiarity, SER, and imageability) when added to the baseline models. Thus, a reasonable question for researchers is whether similar patterns emerge for spaced compounds along similar measures.

Kim et al. (2019) provided an expanded 2,861-word database of unspaced English compound words contained within the ELP. Their database focused exclusively on the role of ST in compound word recognition. ST refers to the relationship between the meaning of a compound word and the meaning of its constituents. For example, the compound word *flagpole* would be considered relatively transparent as it is a *pole* used to fly a *flag*. The compound word *dumbbell*, on the other hand, would be considered opaque, as the compound’s meaning (i.e., a weight used for exercise) cannot be derived from the meaning of its constituents. It should be noted, though, that ST is not an all-or-none variable. In partially opaque compounds, one constituent may contribute directly to the meaning of the compound expression while the other does not (e.g., *crow****bar****, ****jail***bird). As discussed by Kim et al. (2019), and expanded upon below, ST is important for theories of compound word representations and processing. However, the reported impact of ST on actual compound word recognition has been inconsistent in the literature. One possibility for this inconsistency may be because ST has been operationalized differently across studies. For example, Juhasz et al. (2015) provided a single rating of ST on a 1–7 scale for how closely related both constituents were to the compound word’s meaning, whereas Kim et al. (2019) asked participants to rate how semantically related each constituent was to the overall compound word separately, again on a 1–7 scale. Thus, each compound in their database has two transparency ratings, one for the first constituent and one for the second constituent.

The Large Database of English Compounds (LADEC; Gagné et al., 2019) provides the largest database of unspaced English compound words to date. LADEC contains over 8,000 two-constituent compound words that function as nouns. This database again focuses on the ST variable, with participants rating each compound in the database three times. First, they were asked to rate how predictable the meaning of the compound was from both of its constituents on a 100-point sliding scale. Next, they were asked to rate the degree to which the meaning of each constituent was maintained in the meaning of the compound word separately for both the first and second constituent, again on a 100-point scale. In addition to other variables, the LADEC includes corpus-derived measures of ST as well.^2^

These existing compound word databases discussed above (Gagné et al., 2019; Juhasz et al., 2015; Kim et al., 2019) can be used to select stimuli and test hypotheses about unspaced compound words. However, as noted above, concatenation is only one of the three ways to represent compound words in English orthography. There has been growing interest in examining both hyphenated compounds (see Bonin et al., 2022, for a French database) and spaced compounds in English over the past two decades. For instance, Juhasz (2005) explored the processing of English unspaced and spaced compound words across four experiments. The compound words (selected from the CELEX English database; Baayen et al., 1995) were shown in either their correct spatial layout (e.g., *front door*, *softball*) or their incorrect spatial layout (i.e., *frontdoor*, *soft ball*). Participants performed lexical decisions (Experiments 1 and 2) or read the compounds in neutral sentences (Experiments 3 and 4). Across the four experiments, a pattern emerged demonstrating that the insertion of a space between the constituents facilitated decomposition and resulted in faster processing in lexical decisions and on early eye movement measures (first fixation durations). However, the inter-constituent space hindered meaning assignment for the compound word, as evidenced by slower processing for compounds presented with a space in eye movement measures that take refixations into account. Notably, this pattern was similar to a previous finding for German tri-constituent compound words (Inhoff et al., 2000).

One potential limitation of the Juhasz (2005) study is that it treated spatial layout of the compound words as categorical (spaced vs. unspaced). However, there can be variation in how a given English compound word is written. As Kuperman and Bertram (2013) point out, spelling conventions are particularly likely to vary over time, which may result in different preferences across generations. They explored these spelling variations in an analysis of the English Wikipedia corpus. Specifically, they identified 2,306 English noun–noun compounds that were written with different spatial layouts in the corpus. The two most common variations were compounds that alternated between spaced and unspaced formats (984 compounds) followed by those that alternated between spaced and hyphenated formats (856 compounds). The authors computed a spelling bias metric for each compound word, indicating the likelihood of the compound being concatenated. They found that lexical decision and naming times extracted from the ELP for unspaced compound words were affected by spelling bias, with faster responses for compounds that were more likely to be unspaced. This suggests that individuals are sensitive to spelling variation of compound words in English writing; that is, it is harder for an English speaker to accurately respond to a compound word if they typically encounter the word in different spatial formats. Kuperman and Bertram also showed that various properties of the compounds influenced spelling bias. For example, compounds were more likely to be written with a space if they had a longer left constituent, constituents of higher frequency, or greater similarity in meaning between constituents. Conversely, higher-frequency compounds were more likely to be concatenated.

Falkauskas and Kuperman (2015) further explored the impact of spelling bias for English compound words in an eye-tracking study. They embedded words that differed in their bias toward spaced and unspaced formats in neutral sentences and asked participants to read the sentences for meaning. Compounds were presented in both spatial layouts across participants. Similar to Juhasz (2005), they found that initial processing of compounds was faster when presented with a space. Gaze durations and total reading times were longer for compounds presented in a spaced format. However, spelling bias also impacted processing, as it interacted with measures of compound frequency and reader experience. Readers who have more exposure with print (as indexed by the Author Recognition Test; Stanovich & West, 1989) were impacted by spelling bias on the total reading time measure. Likewise, there was a greater effect of spelling bias for high-frequency compound words. In other words, when compound words are experienced more often by a given reader, the reader is more sensitive to their likely spatial layout.

Another line of research has explored how inserting a space into a traditionally unspaced English compound impacts compound recognition (e.g., Elsherif & Catling, 2023, 2024; Elsherif et al., 2020; Frisson et al., 2008; Ji et al., 2011). We will discuss these studies in more detail while highlighting past research on the three rated variables included in the English Spaced Compound Word Database.

## Age of acquisition (AoA)

Rated AoA refers to when a word first enters an individual’s vocabulary and is typically rated by adults based on their subjective experience with words. Rated AoA has been found to impact word recognition across many word processing tasks, including naming pictures, naming words, performing lexical decisions, and reading words in text while eye movements are recorded (for a recent review, see Elsherif et al., 2023). Morrison and Ellis (1995), for example, found that AoA facilitated word naming when participants were asked to read individual words on a screen out loud as quickly as possible. More recently, Catling and Elsherif (2020) reported on a series of picture-naming experiments in which earlier acquired words aided participants’ response times. In another study by Chang and Lee (2020), the authors investigated the role of AoA in an idiographic language (Chinese) compared to an alphabetic one (English). Their study included both a character-naming task and a lexical decision task. While they found that AoA yielded a significant effect on both tasks, it was more influential for the former. These studies converge in demonstrating that earlier acquired words are processed faster than words learned later in life. While many of these studies have focused on morphologically simple words, a growing body of research has shown that AoA is also a strong predictor of compound word recognition time (e.g., Elsherif & Catling, 2024).

Importantly, the utility of AoA as a measure has been challenged (e.g., Bonin et al., 2016; Smolík & Filip, 2022). These arguments have emphasized that AoA is not a direct rating of when specific words are acquired, but rather subjective judgments made by adults whose memories may be confounded by adult word learning and literacy experiences. However, even as a retrospective estimate, AoA ratings provide valuable insights about readers’ attitudes toward specific words, including the pervasiveness of some words during different stages of development. Rated AoA has also been found to have high inter-rater reliability (e.g., Juhasz et al., 2025; Schock et al., 2012) and high criterion validity (Brysbaert, 2016). In addition, AoA has been a strong correlate of word processing outcomes and continues to be an important variable for compound word databases across languages (e.g., Bonin et al., 2022; Zhang et al., 2024).

Juhasz et al. (2015) included AoA as one of the six rated variables in their database of concatenated English compound words. Compound word AoA significantly predicted lexical decision times and naming times extracted for the compounds from the ELP (Balota et al., 2007) when added to a baseline regression model that also included compound word length, compound word frequency and the frequencies of the two constituents. However, the AoA of the compound words’ constituents did not significantly affect processing for these compounds. In a follow-up study, Juhasz (2018) selected 120 of the compound words included in the Juhasz et al. (2015) database and embedded them in neutral sentence frames. Rated AoA for the compound word was found to predict gaze durations and total fixation durations on the compound words when individuals read the sentences while their eye movements were monitored.

Elsherif, Catling, and colleagues (Catling & Elsherif, 2020; Elsherif & Catling, 2021, 2023, 2024; Elsherif et al., 2020), selected a set of more than 200 compound words from the Juhasz et al. (2015) database to systematically explore the role of AoA on the processing across multiple tasks. In Elsherif et al. (2020), the authors found significant effects of whole compound AoA in word naming for compounds presented as unspaced, as well as when a space was inserted between the typically concatenated constituents. Similarly, using the same word list as Elsherif et al. (2020), Elsherif and Catling (2023) report that rated AoA played a larger role in a recognition memory task for spaced compounds than for unspaced compounds. However, they found no difference on a free recall task, suggesting that AoA might influence lower-level processing such as recalling an orthographic cue. AoA also had an impact on both auditory and visual lexical decision times for the same compounds presented in their concatenated forms, as well as with spaces inserted between the constituents (Elsherif & Catling, 2024). Based on these studies, it seems that AoA impacts compound processing when spaces are inserted between the constituents of typically unspaced compound words.

Elsherif and Catling (2021) provide further evidence on this point, investigating the role of AoA, familiarity, and ST (among other variables) on compound processing. Although they tested the same word set as Elsherif et al. (2020), they specifically examined how these variables influenced the processing of each constituent within a compound. Critically, they found that both AoA and ST facilitated readers’ processing of the head (i.e., right-side) lexeme, while AoA and familiarity facilitated processing of the modifying (i.e., left-side) lexeme. This suggests that, while AoA is associated with improved processing for both constituents in a compound word, the role of familiarity in processing the modifying lexeme is more likely the result of explicit parsing rather than automatic parsing. This potential distinction in processing between constituents further supports the need for a spaced compound database that will allow researchers to address these questions. As such, it remains an open question as to whether an AoA effect would be observed for typically spaced compound words. AoA ratings were therefore collected on the compound words included in the English Spaced Compound Word Database to allow researchers to explore the impact of this variable for typically spaced English compound words. In addition, AoA ratings should be useful for developmental researchers in selecting spaced compounds that may be known to children.

## Familiarity

Familiarity is a rated variable that, like AoA, measures an individual’s experience with a given word. However, familiarity is a measure of one’s overall experience over time as opposed to when a word is first acquired. Familiarity has also been found to impact word processing for morphologically simple words in a variety of tasks including word naming and lexical decision (e.g., Balota et al., 2004; Cortese & Khanna, 2007) and recording eye movements during reading (e.g., Williams & Morris, 2004).

Juhasz (2008) noted that English compound words tend to be relatively low in frequency, even for commonly known compound words. Based on this restriction of range issue, it is difficult to design a study that directly explores the impact of compound word frequency on word recognition times for compound words. Given this, Juhasz (2008) used rated familiarity as a measure of subjective frequency in an eye-tracking experiment. Significant familiarity effects were observed on fixation durations for both relatively short (6–7 letters) and relatively long (10 or more letters) concatenated compound words. The effect of rated familiarity was significant in first fixation durations as well as gaze durations, which take refixations into account.

Juhasz et al. (2015) also observed strong effects of rated familiarity for their unspaced compound words on word naming and lexical decision times extracted from the ELP when added to a baseline model that accounted for the length and frequency of the compound as well as the frequency of the two constituents. Familiarity also impacted fixation durations on the subset of the compounds selected for a reading study by Juhasz (2018). Familiarity was found to influence both early (first fixation duration, single fixation duration) and later (gaze duration, total fixation duration) measures. This diverges from the AoA findings reported above, which only showed effects for later fixation duration measures for English compound words, suggesting familiarity has an early but long-lasting effect on reading times for unspaced compound words.

Elsherif and colleagues (Elsherif & Catling, 2021, 2023, 2024; Elsherif et al., 2020) also included familiarity in their research exploring compound word recognition. They observed significant effects of compound word familiarity for all tasks except naming of concatenated compound words and a cross-modal lexical decision. Importantly, familiarity of the entire compound word was significant when spaces were inserted between the constituents in word naming, lexical decision, and memory tasks.

Based on the research reported above, familiarity is an important variable to take into consideration when planning experiments on English compound word recognition. It is particularly useful given the fact that spaced compound words do not appear in commonly utilized sources for word frequency (e.g., Brysbaert & New, 2009). Thus, rated familiarity can provide a measure of subjective frequency of exposure for spaced compound words. Familiarity was therefore included in the English Spaced Compound Word Database to allow researchers to explore the impact of this variable on spaced compound processing and to select items that may vary in their familiarity for native English speakers.

## Semantic transparency (ST)

As discussed above, ST refers to the relationship between the meaning of the constituents and the meaning of the entire compound word. A compound’s constituents may relate to the compound’s meaning in a way that is fully transparent (e.g., *flagpole*), fully opaque (e.g., *dumbbell*), or partially opaque (e.g., *crowbar*; see Libben, 1998, for discussion). ST has been included in theories of compound word representation. According to Libben (1998), transparent compounds are tied to their constituents at both a lexical and semantic level in the mental lexicon, while opaque compounds are only tied to their lexemes at a lexical level. That is, representing a transparent compound like *flagpole* activates the constituents *flag* and *pole* both as word forms and conceptually. On the other hand, an opaque compound like *honeymoon* activates the constituents *honey* and *moon* only as word forms. This idea has gained support from priming studies with English compound words (e.g., Fiorentino & Fund-Reznicek, 2009; Libben, 2003; Marslen-Wilson et al., 1994; Monsell, 1985).

While ST may be theoretically important, its impact on compound word processing in reading and standard word recognition tasks has been mixed. As noted above, Juhasz et al. (2015) found significant effects of rated ST on lexical decision performance but not word naming. Similarly, during a reading task, Juhasz (2018) did not observe an effect of ST on any fixation duration measure when using a subset of compound words selected from the Juhasz et al. (2015) database. Elsherif and colleagues (2020) also did not find effects of ST on word naming performance for either unspaced or spaced compound words. They did, however, observe effects of ST in visual lexical decisions (for both unspaced and spaced compound words) as well as auditory lexical decisions (Elsherif & Catling, 2024). Furthermore, ST affected recall performance for compound words in both spatial presentations (Elsherif & Catling, 2023).

The spatial format of a compound word may contribute to these mixed results concerning ST’s role in processing. Frisson et al. (2008) found no effect of ST on fixation durations when unspaced English compounds were embedded in sentences. However, when a space was inserted between the constituents of the same typically unspaced compounds, an effect of ST emerged in the region after the compound (i.e., the spillover region). Specifically, readers spend more time in the spillover region after reading opaque compounds, indicating that spaced compounds with low transparency may interfere with processing. At a theoretical level, this result suggests that spacing prompts readers to access both constituents of a compound, but this access may impede processing when those constituents are not directly associated with the compound’s meaning. Ji et al. (2011) also found ST effects on lexical decisions for typically unspaced compound words when a space was inserted between constituents, but not when compounds were presented in concatenated form. In their study, participants performed a lexical decision task to compare the processing of compound words to monomorphemic words (e.g., *giraffe*). While they found a processing advantage for both transparent and opaque compound words, this advantage disappeared for opaque compounds when a space was inserted between its constituents. This supports Frisson et al.’s result, suggesting that the inclusion of a space in a compound word enhances access to the compound’s constituents; however, when those constituents do not provide an obvious link to the compound’s meaning, processing is hindered.

Additional evidence comes from a study by Marelli et al. (2015). They investigated the role of distributionally based ST metrics for English compound words that appear in both a spaced and unspaced form (e.g., *home work* vs. *homework*). Through both qualitative and computational techniques, the authors observed that compounds presented in a spaced format were more directly linked to semantic neighbors tied to the literal interpretations of the constituents. For example, the spaced compound *home work* was associated with words like *house* and *job*, while the unspaced *homework* was associated with *school* and *class.* They concluded that the relationship between a compound word and its constituents can vary based on the spatial format of the compound word, further justifying the need to investigate ST across different compound formats. Furthermore, in terms of processing implications, Marelli et al. report that the measure of ST based on the spaced format of the compound was also more effective in capturing variance in lexical decision times from the ELP relative to the measure based on the concatenated form of the same compound word.

Given the theoretical importance of the ST variable, we have included ratings of this variable in the current database. While many options for ST metrics exist, we have chosen to use the 1–7 scale utilized by Juhasz et al. (2015) to facilitate comparison between the current database and the unspaced compound words contained in Juhasz et al. (2015). Notably, however, researchers have conceptualized ST in various ways, which has likely contributed to the mixed results found for this variable (Auch et al., 2020; Gagné et al., 2016). While some studies have operationalized ST via the predictability of a compound’s meaning from its constituents (Juhasz et al., 2015), other studies have simultaneously explored the role of meaning retention for a constituent within a compound (e.g., Gagné et al., 2019; Marelli & Luzzati, 2012). To illustrate this difference, consider the compound word *horseshoe*. Under a predictability rating, participants would be asked to rate how predictable the meaning of *horseshoe* is given the constituents *horse* and *shoe*. Conversely, under a retention rating, participants would be asked to rate how well the meaning of both *horse* and *shoe* are preserved in the compound *horseshoe*. Although subtle, the different rating scales carry different implications about the theoretical constructs under discussion. Additionally, these ratings carry different implications from other operationalizations of this variable, including human and computational assessments (see Auch et al., 2020, for discussion). Despite these variations, we have elected to include only the predictability variation of this rating in the present database, given this rating’s consistency across other compound databases (e.g., Juhasz et al., 2015), as well as its strong correlation with retention ratings (especially for the first constituent; see Auch et al., 2020) and reliability in predicting lexical decision times from databases like the ELP.

## Spaced compound word processing and its theoretical motivation

While compound words have been used as stimuli to explore morphological processing and word recognition, they have also been useful for exploring issues related to eye movement control during reading. Notably, a debate exists in the eye movement and reading literature as to whether reading is a serial process, wherein words are attended to and lexically identified one at a time (e.g., E-Z Reader model and the more recent Über-Reader model: Reichle, 2011, 2021; Reichle et al., 1998), or a parallel process wherein multiple words can be lexically identified simultaneously during a single fixation (e.g., SWIFT model and more recently, the SEAM model: Engbert et al., 2002; Engbert & Kliegl, 2011; Rabe et al., 2024; see also Zang, 2019, for a discussion). Given that the identification of a word in the lexicon is considered a primary determinant of when, and where, to move the eyes from the current fixation to the next, it should be apparent that the question of whether lexical identification occurs serially and sequentially, or in parallel, is critical in respect of formal models that account for such decision-making. Of course, in English (and other spaced alphabetic languages), while it may initially appear straightforward to consider letter strings that are visually separated by spaces as being a basic lexical unit—that is, a word—it should be apparent from the preceding discussion that such an assumption may be overly simplistic.

Indeed, a recent theoretical account, the Multi-Constituent Unit (MCU) Hypothesis (Zang, 2019; Zang et al., 2021; Zang et al., 2024a, 2024b; see also He et al., 2021), proposes a more nuanced possibility. According to the MCU Hypothesis, as per standard accounts of lexical processing, individual words are stored in the lexicon. However, alongside these representations, there are also lexicalized representations for units that comprise multiple words with a spaced format, and these are referred to as multi-constituent units (MCUs). To be specific, linguistic units such as common spaced compound words, idioms, frequently occurring phrases, real names may be represented lexically as unified lexical representations. Thus, according to the MCU Hypothesis, the form that a lexical unit takes can be flexible and dynamic, in the context of reading across spaced (and unspaced) alphabetic (and nonalphabetic) writing systems (see Zang, 2019).

Importantly, in the context of the serial/parallel debate associated with current eye movement control models in reading, experimental evidence indicating that multiple words are lexically identified in parallel is compatible with parallel eye movement control models (e.g., SWIFT, SEAM); however, it poses a serious challenge to strictly serial models (e.g., E-Z Reader, Über-Reader). If frequently co-occurring strings such as spaced compounds were represented as single unified lexical representations (MCUs), and these were processed as such during natural reading, then evidence of parallel processing across the constituents of the MCU would not violate assumptions of seriality of lexical identification. That is, serial lexical processing might occur on a lexical unit by lexical unit basis, but some of those units may be MCUs and represent more than one word. Regardless of the details of the MCU Hypothesis specifically, what should be apparent from this discussion is that the way lexical units are defined, and how processes associated with their identification are operationalized over those units during natural reading, has important implications for theoretical accounts of eye movement control.

Compound words can provide a useful test for the extent of serialism versus parallelism, as they are single conceptual units that are composed of multiple constituents. Researchers have manipulated the parafoveal preview of the second constituent in unspaced compound words (e.g., Drieghe et al., 2010; Juhasz et al., 2009) as well as spaced compound words (e.g., Cutter et al., 2014; Juhasz et al., 2009) to explore how effectively readers process the upcoming constituent before it is fixated. And, indeed, these studies have delivered results that are consistent with the MCU Hypothesis (see also Zang et al., 2021, 2024a, 2024b, for further evidence for MCU processing in Chinese).

As one example, Cutter et al. (2014) manipulated the parafoveal preview of the first and second constituent of English spaced compound words (e.g., *teddy bear*, *scuba diving*) using the boundary paradigm. This paradigm can determine how much information readers are able to access about upcoming words in the parafovea. Prior to fixating on the compound word, the constituents were either fully available in the parafovea or masked with random letters, resulting in four preview conditions (e.g., *teddy bear; teddy hocu; fohbg bear; fohg hocu*). These parafoveal previews were always replaced with the actual spaced compound word when it was directly fixated. Cutter et al. demonstrated that readers gained parafoveal preview of the second constituent of the spaced compounds if the first constituent was also available for preview in the parafovea. This suggests that readers process the two constituents of spaced compounds as part of a larger lexical item during reading and are therefore able to flexibly adjust their perceptual span to preview the second constituent. Given the interest in investigating how spaced compound words are lexically processed, it is very likely that a database of such words may be useful for facilitating selection of stimuli for future experiments. This was one of the motivations for us in developing the English Spaced Compound Word Database.

## The English Spaced Compound Word Database

The purpose of the present study is to provide a database of English spaced compound words for researchers to select items and conduct analyses to answer questions related to word recognition and reading processes. The database includes 1,162 two-constituent spaced compound words which were selected from the Webster’s Dictionary for Students (6th Ed.). Although other databases have gleaned word lists from naturally occurring speech or text (e.g., SUBTLEX-US; Brysbaert & New, 2009), we elected to consult a dictionary for several reasons. First, there is precedent for using a dictionary as a source for large word databases (e.g., CELEX; Baayen et al., 1995), establishing its utility as an authoritative guide for both lexical entries and spelling conventions. Second, the use of a student dictionary, specifically, should facilitate researchers at universities who principally recruit student-age participants for compound word studies. Finally, analysis of a dictionary, as opposed to a naturally occurring text source, offers an objective guide in determining what should and should not count as compound words. One challenge with analyzing spaced compounds is determining what word pairs constitute an actual compound word as opposed to a mere collocation of two words. In naturally occurring sources, every two-word string could theoretically be counted as a spaced compound; otherwise, the researchers would have to rely on the presence of some specific feature or an arbitrary frequency cutoff to determine what counts. Thus, by using a dictionary, we are limiting our database to word pairs that have established conceptual representations in the language. Under this guideline, we considered all two-word entries in the dictionary for the English Spaced Compound Word Database. Furthermore, we did not discriminate between compound words based on their constituents’ part of speech. Although prototypical unspaced compounds are headed by a noun and modified by either a noun (e.g., *paperclip*) or adjective (e.g., *blueberry*), unspaced compounds can also constitute closed class grammatical items (e.g., *hereby, onto, before*). Thus, for our collection of spaced compounds, we considered all of the dictionary’s spaced entries. Properties of the compound words’ constituents are included from other available databases, including length, frequency, AoA, and concreteness (among others). In addition, the database includes newly collected ratings for AoA, familiarity, and semantic transparency for the entire spaced compound expression.

## Method

### Participants

We recruited 183 participants through Prolific (104 female, 76 male, 3 non-binary), ages 18–74 years (*M* = 37.4, *SD* = 12.9), who completed the study between November and December 2024. Each spaced compound was therefore rated by approximately 30 participants. This is an increase in number observations compared to Juhasz et al. (2015) and is in line with other recent compound word databases (e.g., Bonin et al., 2022; Kim et al., 2019). Prolific filtered participants prior to testing to include only those who registered as native English speakers and were located in the United States at the time of accessing the experiment. Additional prescreening conducted by Prolific verified that all recruited participants self-reported learning English between the ages of 0 and 4 (*M* = 1.4, *SD* = 1.2). The sample was further restricted to include only participants with an established positive track record on Prolific (> 95% success rate). Prolific automatically removed participants from the sample if they exceeded Prolific’s maximum time allowed to complete the study (> 87 min. on an estimated task completion time of 30 min). Any participants who did not meet this eligibility criteria were prohibited from accessing the study entirely, and this process was managed by Prolific. Upon completing the study, we performed checks to verify whether the eligible participants completed the study in good faith, excluding 33 participants who failed the majority of attention check items (see below).^3^ The study continued to run until a full sample of at least 60 eligible participants per rating condition was obtained, resulting in the full sample of 183 participants. Due to concerns over formatting inconsistencies, respondents were restricted from participating if they accessed the study via a mobile phone. Participants were compensated $15 for their responses. Average completion time was 36 min 24 s (*SD* = 15.5 min).

### Materials

We compiled a list of spaced compound words using Webster’s Student Dictionary (6th Ed.) by manually selecting all multi-word entries (*n* = 1,294) from approximately 37,000 total entries. We then removed individual entries for one of the following reasons: proper nouns (e.g., *Santa Claus*; *n* = 79); duplication^4^ (e.g., *chicken out* vs. *chickened out* vs. *chickening out*; *n* = 26); loan words (e.g., *et cetera*; *n* = 15); excessive constituents^5^ (e.g., *sports-utility vehicle*; *n* = 10); numeric entries (e.g., *2 dues*; *n* = 2). All remaining words were included in the final list (*n* = 1,162). Due to concerns about completion times and concentration loss, we decided to create two distinct lists. To do this, we alphabetized the full word list and then sorted every other word into a distinct list as a way of partitioning similar words (e.g., *half brother* and *half sister* appear on separate lists). The full word list and all corresponding data and analyses are publicly available at https://osf.io/65prn.

#### Constituency profile

We tagged each constituent’s part of speech using the ELP’s preexisting tags. However, in a number of cases, these tags proved insufficient. For instance, the ELP tags do not differentiate among what it considers “minor” parts of speech, such as prepositions and determiners. Additionally, there were several words that did not exist in the corpus and were not tagged. In these cases, we manually tagged the entries. Another concern stems from the fact that many words can be considered as multiple parts of speech (e.g., *can* may be used as either a verb or noun). In cases where the ELP listed multiple parts of speech for a word, we accepted the first entry, as this represented the most frequent tagging. However, in numerous cases, the primary part of speech listed did not align with the semantic sense of the word being tagged. Thus, we manually tagged items whose ELP tags did not match our best judgment. A more detailed breakdown of this process can be obtained by referencing our database: https://osf.io/65prn.

The grammatical profile of the compound word list is provided in Table 1. Comparing parts of speech, we see that the database is dominated by noun–noun, modifier–noun, and verb–preposition combinations. As mentioned above, we considered all multi-word entries from the dictionary to provide a definitive account of which word pair co-occurrences should be included in the database, as each pairing bears a distinct conceptual representation and may form the basis of future unspaced compounds. However, we do want to acknowledge that the various parts of speech may lead to differences in processing due to the syntactic structures that they constitute. For example, multiple behavioral tasks have found processing deficits for verbs compared to nouns, with more striking differences in aphasia cases (e.g., Luzzatti et al., 2002). It is also worth pointing out that not all collocations compound in the same way. The prevalence of noun-based compounds in our database suggests that nouns may be commonly grouped together or with other parts of speech to form new concepts. For other parts of speech, however, this may be more difficult to sustain. For instance, English does not typically encode direction or specific aspects of motion directly into the verb; rather, English relies on particles to express these semantic features (e.g., *move out*, *march on*, *drive up*). Talmy (1985) describes these particles as “satellites” due to the flexibility of their placement in a sentence (e.g., *We drove up to my parent’s house in Michigan* vs. *We drove to my parent’s house up in Michigan*). Thus, the dynamic nature of these particles may make these phrases more resistant to conventionalization as compounds.

**Table 1:** Part-of-speech profile for spaced compound word entries

First constituent	Second constituent	Number of entries	Example
Interjection	Noun	1	*aha moment*
Modifier	Noun	265	*bald eagle*
Modifier	Preposition	9	*black out*
Modifier	Verb	2	*intensive care*
Noun	Modifier	3	*notary public*
Noun	Noun	475	*car seat*
Noun	Preposition	21	*chalk up*
Noun	Verb	10	*milk shake*
Possessive	Noun	3	*athlete’s foot*
Preposition	Modifier	8	*in vain*
Preposition	Noun	44	*at length*
Preposition	Preposition	12	*inside out*
Preposition	Verb	3	*for rent*
Verb	Modifier	10	*get even*
Verb	Noun	74	*dodge ball*
Verb	Preposition	217	*blow over*
Verb	Verb	5	*make believe*

We then compiled the characteristics of each constituent that made up the compound words in the study (see Table 2 for a descriptive summary). We first calculated word length, syllable count, and morpheme count for each constituent via the ELP. In a few cases, we manually corrected inaccurately reported values (e.g., we used a character-counting function to check word lengths). With the ELP, we then determined the orthographic Levenshtein distance (OLD), concreteness, and AoA of each constituent. OLD represents the average orthographic distance between a given word and its 20 nearest orthographic neighbors. Smaller OLD values therefore represent greater orthographic similarity. Concreteness is represented as a mean rating for each word that measures how perceptible an entity is by its corresponding word (Brysbaert et al., 2014). For example, *chimney* is considered to have high concreteness, while a more abstract term like *dream* is not. AoA is represented as the average age at which people judge themselves to have learned a given word (Juhasz, 2005; Kuperman et al., 2012). These data were available for nearly all constituents.

**Table 2:** Descriptive summary of spaced compound constituents included in the ratings task. Data were compiled from the ELP and the SUBTLEX-US corpus

Rated variable	Number of constituents	Min. range	Max. range	Mean	*SD*
Length	2,324	2	15	4.95	1.94
Syllables	2,324	1	6	1.52	0.77
Morphemes	2,324	1	5	1.21	0.48
OLD	2,280	1.00	6.30	1.76	0.63
Concrete	2,225	1	5	3.76	0.97
AoA	2,253	2.37	15.79	6.01	2.50
Freq (wpm)	2,315	0.02	22,677.84	876.20	2,440.20
Log freq	2,315	0.30	6.06	3.43	1.16

We then determined the frequency and log frequency of each constituent via the SUBTLEX-US corpus, a public database of English words from television and movie subtitles (Brysbaert & New, 2009). The corpus defines frequency as the occurrence of a word per million words (wpm), and log frequency as the log_10_ of a word’s frequency + 1. All but nine of the constituents for which we collected ratings were included in the database, presumably due to their rarity: *aleck*, *bituminous*, *caddis*, *cecropia*, *diacritical*, *maidenhair*, *Komodo*, *ordinal*, and *responder*.

### Procedure

Eligible participants accessed the study through Qualtrics software (Provo, UT). Participants were randomly assigned to one of the two word lists, and then randomly assigned to one of three tasks: familiarity, AoA, or ST. Borrowing from Juhasz et al.’s (2015) methodology, participants were asked to provide a 1–7 rating for each task. The specific instructions from each task are included in the online repository for this database (https://osf.io/65prn). In the familiarity task, participants rated how familiar each compound word (on their assigned list) was to them. We defined familiarity as knowing a word’s meaning and using it frequently. For example, a commonly used word like *the* should be rated as more familiar than a seldom used word like *quotient*. Participants rated each word’s familiarity on a scale of 1 (not familiar at all) to 7 (very familiar).

In the AoA task, participants were asked to rate each compound word on when they first learned its meaning in either spoken or written form, using the same instructions as several previous studies that measured AoA (e.g., Gilhooly & Logie, 1980; Juhasz et al., 2015). An example of a word that is acquired early in life might be *count*, while an example of a word acquired later in life might be *algebra*, corresponding to both a difference in frequency and age-appropriateness. Because participants may not have direct access to knowledge about when they learned a word, they were asked to provide a post hoc approximation (see Juhasz, 2005; Kuperman et al., 2012). The ratings again used a 1–7 scale (first developed by Gilhooly & Logie, 1980), though with each value explicitly assigned to an age range: 1 (age 0–2); 2 (age 2–4); 3 (age 4–6); 4 (age 6–8); 5 (age 8–10); 6 (age 10–12); and 7 (age 13 +).

For the ST task, we chose to ask participants to rate each compound for how transparent the meaning of its constituents was to the compound’s overall meaning. These instructions represent one of several ways to measure ST (see discussion above). For example, *green bean* is a compound word with high ST because the compound’s meaning is transparent from the meaning of its constituents individually (i.e., the meaning corresponds to a bean that is green). Meanwhile, *green thumb* is a compound word with low ST because the compound’s meaning is not transparent from the meaning of its constituents individually (i.e., the meaning does not directly correspond to *green* or *thumb*). Once again, the task included a scale of 1 (not transparent at all) to 7 (very transparent) for ratings.

All words from a participant’s assigned list were presented on a single screen in randomized order. The words were divided into groups of 10 for easier visual processing, and to allow participants access to the ratings scale without having to scroll to the top of the page. Although we did not explicitly inform participants that they could skip rating individual words, they were not forced to provide a rating for every compound word that they saw. In addition to the 581 words on each list, 7 attention check items were included to ensure that participants were engaging with the task. These items consisted of nonce words that were orthographically constructed to appear as spaced compounds (e.g., *crinby warky*). Participants were instructed to rate these items in a manner that was accurate within their assigned task (i.e., a rating of 1 on familiarity and ST tasks, and a rating of 7 on the AoA task.

## Results and Discussion

All 183 eligible participants were randomly assigned to one of the three ratings tasks and to one of the two word lists, resulting in at least 30 participants per condition. No participants were excluded from analysis, nor were any individual responses removed from the data. Because participants could skip trials if they wished, only 105,921 observations are recorded of a possible 106,506. The use of a 1–7 scale for ratings limited the range of responses, preventing any one response from being too extreme in either direction. Although word ratings varied, we wanted to include a diverse set of items, so we did not exclude any individual words from analysis. We report descriptive statistics for each task in Table 3. The mean ratings for AoA and ST were normally distributed, and the mean ratings for familiarity were skewed toward high familiarity. Interrater reliability was quantified for each condition via an intraclass correlation coefficient (ICC). ICCs were analyzed in R (v4.3.3; R Core Team, 2024) using the *psych* package (v2.5.3; Revelle, 2025).^6^ Reported coefficients for each condition, ICC(3,*k*), correspond to the data structure; that is, ICCs of a two-way mixed effect model that relies on mean ratings from multiple raters to determine ratings’ general consistency with one another (Koo & Li, 2016). For all six conditions, ICC > .90, indicating strong interrater reliability.

**Table 3:** Descriptive summary of rating tasks for spaced compounds across both word lists

Task	*N*	Min. avg. rating	Max. avg. rating	Avg. mean	*SD* of mean
Familiarity (all)	62	1.65	6.97	5.84	1.14
*List 1*	*31*	*1.65*	*6.97*	*5.83*	*1.38*
*List 2*	*31*	*1.65*	*6.97*	*5.85*	*1.32*
AoA (all)	61	1.61	7.00	4.68	1.23
*List 1*	*31*	*1.61*	*6.93*	*4.51*	*1.38*
*List 2*	*30*	*2.23*	*7.00*	*4.85*	*1.37*
ST (all)	60	1.93	6.53	4.22	1.01
*List 1*	*30*	*2.00*	*6.53*	*4.41*	*1.88*
*List 2*	*30*	*1.93*	*6.13*	*4.02*	*1.95*

For analyses, we planned to examine the correlations between each rating task overall (i.e., AoA vs. familiarity; AoA vs. ST; familiarity vs. ST). To do this, we first calculated the average rating for each word in the database. We then calculated a Pearson correlation coefficient to assess the relationship between rating tasks (α = .05). We analyzed the data in R with the basic *stats* package using the cor.test function (R Core Team, 2024). We found a strong negative correlation between familiarity and AoA, *r*(1,162) =  − .70, *p* < .01, as well as a positive correlation between familiarity and ST, *r*(1,162) = .22, *p* < .01. The correlation of AoA and ST was also statistically significant but weak enough to be practically irrelevant, *r*(1,162) =  − .06, *p* = .04. The strong negative correlation between familiarity and AoA suggests multicollinearity between these variables; thus, future research should control for this relationship when considering the independent predictability of these variables.

### Descriptive characteristics

As reported in Table 3, the compound words ranged in average familiarity, AoA, and ST. We then evaluated the relationship between compound words and their constituents. Although data on the descriptive characteristics of whole spaced compounds were generally unavailable, we explored the correlation between these compounds’ ratings and the descriptive characteristics of each of their constituents in Table 4. To do this, we considered the average rating for each word on all three of our ratings tasks and performed individual correlational analyses between each rating task and each of the following descriptive characteristics: character length, number of syllables, number of morphemes, OLD, concreteness, AoA, frequency, and log frequency. These values were obtained from the ELP (see Table 2). We again conducted our Pearson correlation analyses in R using the cor.test function. While correlations between the rating tasks and constituent properties are discussed below, two observations stand out. First, the relationship between the rating tasks and a constituent’s concreteness was weak (*r* < .20 in 5 of 6 tasks), particularly with spaced compounds’ AoA ratings. Second, the relationship between ratings and a constituent’s complexity—i.e., its character length, syllable count, number of morphemes, and OLD—is weaker for second constituents across tasks. That is, when constituents are more complex, the association with a corresponding spaced compound’s familiarity, AoA, and ST appears stronger for constituents in the earlier position. One exception, though, is the relationship to constituents’ number of morphemes, which is weakly correlated with all measures for both constituents.

**Table 4:** Pearson’s correlation coefficient *r* for mean spaced compound word ratings on all three ratings tasks and the descriptive qualities (see Table 2) of each constituent separately (C1 = first constituent; C2 = second constituent). All values are statistically significant (*p* < .001) except for coefficients between familiarity-C2 and number of morphemes (*p* = .088) and between AoA-C1 and concreteness (*p* = .053)

Task-constituent	Len	Syll	Morph	OLD	Concrete	AoA	Freq	LogFreq
Familiarity-C1	− .26	− .25	− .12	− .23	− .13	− .30	.18	.40
Familiarity-C2	− .17	− .12	− .05	− .15	− .19	− .22	.17	.35
AoA-C1	.34	.31	.19	.33	.06	.46	− .19	− .44
AoA-C2	.30	.21	.17	.22	.14	.42	− .24	− .43
ST-C1	.30	.27	.23	.29	.18	.20	− .25	− .27
ST-C2	.32	.16	.13	.15	.40	.23	− .34	− .39

Additionally, we evaluated the relationship between the rated variables of familiarity, AoA, and ST with the length of the spaced compound word as a whole unit. Word length was determined via a character counting function for each word, ignoring spaces and punctuation. We then conducted a Pearson correlation analysis to assess the relationship between total length and the rated variables. We found a significant negative correlation between length and familiarity, *r*(1,160) =  − 0.28, *p* < .001, and positive correlations between length and AoA, *r*(1,160) = 0.41, *p* < .001, as well as length and ST, *r*(1,160) = 0.23, *p* < .001. Notably, each of these is similar to the correlations between the rated variables and the lengths of the compound words’ constituents reported in Table 4.

### Demographic profiles

In addition to the spaced compound word ratings, we collected a limited amount of demographic information from participants, consistent with what is minimally required for reporting (American Psychological Association, 2020). This included participants’ self-reported age, gender, and race. We also asked participants to report the age at which they began speaking English and whether they spoke any other languages. All reported information is available in the database.

Using this information, we conducted a post hoc exploration of whether there was any relationship between the demographic factors of age, gender, and race and participants’ responses. We observed only weak relationships between age and ratings (AoA: *r* =  − 0.13; familiarity: *r* = 0.15; ST: *r* =  − 0.19). There were no significant influences of gender identity or racial identity on word ratings. Thus, it appears that the ratings in our database are largely unaffected by specific demographic information, at least on the three factors considered here.

### Familiarity

Spaced compound word ratings skewed toward higher familiarity, similar to what was found previously with unspaced compound words (Juhasz et al., 2015). Of the 1,162 compounds, 760 (65.41%) averaged a rating above 6. The five words rated as most familiar were *French fry*, *human being*, *shut up*, *fast food*, and *rubber band*, while the least familiar were *cecropia moth*, *prairie schooner*, *alimentary canal*, *bituminous coal*, and *spring peeper*. Intuitively, these compounds seem to align with their general frequency; however, consistent with Juhasz et al., the relationship between familiarity and constituents’ frequency was low (*r* < .20), though statistically significant. Thus, the more frequent a constituent, the more familiar the compound.

Beyond frequency, familiarity of spaced compounds correlated negatively with all other of the ELP’s descriptive variables for both constituents. As mentioned above, highly familiar compounds were more likely to coincide with less complex constituents (i.e., shorter character lengths, fewer syllables, fewer morphemes, and smaller OLD values), especially for the first constituent, though this relationship was weak overall. The negative direction of these values supports Zipf’s (1949) observation that more frequent words are systematically shorter in length than less frequent words (to the extent that constituent frequency and the compound’s overall familiarity correlate). For spaced compounds, this relationship may be especially revealing, as each constituent’s complexity is roughly doubled by the presence of the other constituent; thus, a compound consisting of two high-complexity constituents suggests that a reader is likely to be less familiar with the compound word as a whole. This relationship is also consistent with the negative correlation of familiarity to each constituent’s AoA, suggesting that more familiar compounds are more likely to consist of constituents acquired earlier in life. Juhasz et al. (2015) report a similar finding for unspaced compounds, confirming the close bond between these two variables. Taken together, these findings indicate that readers are more familiar with spaced compound words that are composed of constituents that are also highly familiar. In conjunction with being highly familiar, these constituents tend to consist of simpler structures (shorter lengths, fewer syllables, and fewer morphemes), as well as being more similar to other words (as measured by OLD), more frequent, and acquired earlier.

Finally, as mentioned above, the correlation between familiarity and concreteness was weak enough that it is unlikely to be practically relevant. Still, the negative coefficient for this relationship suggests that readers may be more familiar with compounds that consist of more abstract constituents, a contrast from Juhasz et al.’s (2015) previously reported relationship between familiarity and overall compound word imageability (*r* = .48) in unspaced compound words. They defined imageability as the degree to which words evoke a visual representation, which is roughly equivalent to the concreteness measure that we have included. The lack of association in the current study may be due to a difference between unspaced and spaced compounds. For instance, entities that are highly imageable may hold stronger memory representations which, in turn, facilitate the compounding process (see Kuperman & Bertram, 2013). For spaced compounds, this relationship may simply be weaker on average. In addition, the correlations calculated in the present study are between the concreteness of the constituents as opposed to the concreteness of the entire spaced compound. Future research could further explore semantic variables for the entire spaced compound word (e.g., concreteness, imageability, and sensory experience ratings), which was beyond the scope of the present study.

### Age of acquisition

While AoA responses varied across words, recall that higher AoA ratings represented judgments of later acquisition. Average responses were normally distributed, with the overall average (*M* = 4.68; see Table 3) corresponding to acquisition at roughly age 7. The five compounds with the lowest average AoA were *teddy bear*, *ice cream*, *belly button*, *lay down*, and *at home*, while the five with the highest average AoA were *cecropia moth*, *cloud computing*, *airman basic*, *maidenhair fern*, and *alimentary tract*. One qualitative observation from the data is that compounds associated with stereotypical childhood events (e.g., *teddy bear*, *coloring book*, *nursery rhyme*) had some of the lowest AoA ratings. While early exposure to compounds is not necessarily equivalent to frequency, AoA ratings were negatively correlated with the frequency of both constituents in the ELP (as well as familiarity rating of the whole compound word); that is, less frequent constituents were more likely to be components of a compound acquired later in life.

As reported in Table 4, AoA ratings for spaced compounds were positively correlated with AoA ratings for each of the compound’s constituents in the ELP (*r* > .40). While this relationship may seem obvious, these coefficients may offer insight into the language acquisition process. For instance, the early acquisition of some words may facilitate one’s ability to interpret those words in context, including knowledge about collocations that are habitually compounded (e.g., Riches et al., 2022; Smith & Murphy, 2015). In cases of conventionalization (e.g., *teddy bear*), it seems plausible that children might acquire the compounded form before at least one of the individual constituents (i.e., *teddy*), but in general it appears that constituents acquired early may lead to compound forms which are also acquired relatively early. In the same vein, AoA ratings were positively correlated with complexity (i.e., longer character lengths and more syllables and morphemes). This finding is consistent with the negative correlation of complexity and familiarity such that people are likely to encounter simple, shorter words early in life and quickly integrate those words into their lexicons (e.g., Ellis, 2012). The positive correlation between AoA and OLD also supports this view of acquisition; that is, constituents with more orthographic competitors in proximity are more likely to be judged as having been acquired later within a compound. This is likely because discerning these forms from many similar words is challenging to readers (Grainger et al., 2016). Notably, this relationship appears to contrast with one reported by Storkel (2024) on the relationship between AoA and phonological neighborhood density. This may highlight a distinction between the functional roles of phonological and orthographic density, as children presumably interact with words’ orthographical features less often than their phonological features, given that they generally receive verbal, but not written, input consistently from birth (Gough & Hillinger, 1980).

Finally, we report only a weak positive correlation between AoA spaced compound word ratings and the concreteness of their constituents according to the ELP. This suggests that the concreteness of a word plays only a minor role in the word’s ability to function as part of a compounded form. As with familiarity, this is contrary to what one might expect considering the relationship of unspaced compounds and imageability, as well as of unspaced compounds and SER (Juhasz et al., 2015). Additionally, concreteness is thought to facilitate acquisition of nouns before verbs in children (Gentner, 1982), though this point is debated (Childers & Tomasello, 2006; Nelson et al., 1993; see Waxman et al., 2013, for a review). The lack of correlation here may be a partial consequence of the nature of spaced compounds. That is, because spaced compounds consist of two orthographically distinct lexemes, the role of concreteness in each lexeme may not be as critical to their interpretation as it is for processing the compound overall.

### Semantic transparency

Average ST ratings were normally distributed. Spaced compounds with the highest average ST ratings were *steering wheel*, *flowering plant*, *apartment building*, *lawn mower*, and *letter carrier*, while the lowest ratings were *by turns*, *hot rod*, *witch hazel*, *palm off*, and *bowl over*. These ratings appear to align with reality; for example, a *steering wheel* is literally a wheel that one uses for steering, while a *hot rod* is literally neither hot nor a rod. Thus, it appears that participants rated idiomatic and conventionalized figurative uses of language as less semantically transparent (see Benczes, 2005). Like the AoA ratings, ST ratings are negatively correlated with the frequency of their constituents in the ELP, implying that less frequent words make for constituents that are more transparent to a compound’s meaning. One explanation for this is that less frequent words may be less likely to be used in ways that deviate from their literal meaning (Kuiper et al., 2018). For example, *steering* may be used less frequently than *hot*, but the semantic range of *steering* is more limited as a result. Because *hot* has many different meanings that can be used across many contexts, its frequency is likely to be higher; however, the trade-off of being more versatile is that its intended meaning in a particular context becomes less transparent (Griffin, 1999). This explanation is also consistent with the finding that ST is positively correlated with the complexity of each constituent’s word form according to the ELP measures, especially character length. Because longer words are less flexible in their usage, they tend to constitute compound words that are more semantically transparent. Similarly, when constituents—especially the first constituent—had more syllables, more morphemes, and greater orthographic density, the compound words composed of these constituents were more likely to be judged as semantically transparent.

Concreteness also appears to influence constituent order. Although constituents’ concreteness was weak for familiarity and AoA compared to other properties, the correlation with ST is relatively strong, especially for the second constituent (*r* = .40). Because many spaced compound words are endocentric (i.e., they include a head noun as in *steering wheel*, *flowering plant*, *stringed instrument*, etc.; see Bisetto & Scalise, 2005), at least one tangible referent should be available in most cases. As Gagné and Spalding (2008) point out, the head of a compound often occurs in the second position, contrary to common morphological patterns found throughout the language, and this explanation is consistent with the strong correlation of concreteness in the second constituent reported here. Finally, a compound’s ST was also positively associated with its constituents’ AoA such that constituents that were judged to have been acquired later in life constituted compounds that were deemed more transparent. One possible explanation for this association is that many of the words that make up spaced compounds with low ST are closed-class words (e.g., prepositions as in *flat out*). While these kinds of constituents may be functional and more likely to be acquired earlier in life, their meanings are more ambiguous and less concrete.

## Conclusion

The ratings reported in the English Spaced Compound Word Database reflect readers’ average experience with a wide variety of English spaced compound words. The choice to examine spaced compound words serves two major purposes. First, for practical reasons, the ratings provide researchers with a database of spaced compound words, readers’ experiences with those words, and reported characteristics of the constituents that make up those words. This establishes a baseline from which to select stimuli for future study, especially for word recognition and reading tasks. While interest in compound word research has increased in recent years (e.g., Kuperman & Bertram, 2013), this work has largely centered on unspaced compounds, or the alternation between spaced and unspaced forms of singular concepts (Juhasz, 2005). Spaced compound words add a complicating factor for researchers, namely, addressing the boundary between a spaced compound word and two words that merely co-occur. This gradient boundary contributes to spaced compound words being more difficult to define than unspaced compound words. Thus, a major goal of this paper was to rely on the spaced compound entries of an authoritative source (i.e., a dictionary) and provide readers’ reported experiences with those words. It is important to note that no database of processing times for English spaced compound words currently exists. Therefore, to our knowledge, this database represents the only existing set of rated spaced compound words, serving as a starting point for researchers interested in these words. It is our hope that researchers will use this database to select items to include in studies exploring how English spaced compound words are processed using tasks such as reading, word naming, and lexical decision.

Second, for theoretical reasons, the data collected here provides insight into readers’ experiences with spaced compound words, fostering speculation about the nature of readers’ mental lexicons and how lexical processing operates over linguistic units during natural reading. We noted the importance of compound words (among other multi-word units) to current accounts of processing such as the MCU Hypothesis. Like other compound word forms (i.e., unspaced and hyphenated), spaced compound words conjoin two distinct concepts into a single lexical entry; however, the orthographic space between constituents allows spaced compounds to maintain the surface-level appearance of two distinct lexical items. Understanding how readers engage with spaced compound words relative to unspaced compound words and non-compound words is essential for determining the organization of the lexicon. For instance, a low-ST compound word that is unspaced (e.g., *pineapple*) is less likely to mislead readers into interpreting the word as the sum of its constituents than a low-ST compound word that is spaced (e.g., *hot rod*). As readers encounter spaced compound words, they are likely to access both the compound’s meaning and the meaning of both constituents. In high-ST cases, this should facilitate comprehension and strengthen the conceptual ties between the compound and its constituents; however, in low-ST cases, decomposing the compound word into its parts could be detrimental to comprehension. As mentioned above, comparing spaced and unspaced compound words is confounded by the fact that compounds often become conventionalized as unspaced through frequent use; however, the conventionalization process may also contribute to an increased semantic distance between a compound and its constituents.

Notably, the ratings collected in the English Spaced Compound Word Database reflect a variety of words that range from high familiarity, high transparency, and/or early acquisition, to low familiarity, low transparency, and/or late acquisition. These three measures provide a well-balanced context for readers’ encounters with spaced compound words. In particular, the correlation between these measures confirms a tight relationship between familiarity and AoA, such that the words that readers acquire early in life are often the ones with which they report being most familiar. These ratings may also inform on sociocultural differences in people’s experiences with words, as our sample reflects the judgments of Americans who are native English speakers. Regional or other interpersonal differences may exist regarding specific compounds (e.g., older readers may be more familiar with compounds like *answering machine* than younger readers, or *cayenne pepper* may be more familiar to readers in regions that have more access to this spice). Importantly, readers’ attitudes toward and experiences with these words are certain to differ in other languages, particularly those that use different compounding methods or draw different semantic boundaries on familiar concepts. Other limitations include the methodological choices by which we evaluated the spaced compounds in the database. For instance, the ratings we collected are based on between-subject accounts of participants’ subjective experiences with individual words. Additionally, our ratings were collected using online data collection, and while we took steps to ensure the validity of our sample, this environment allows for less experimental control than in-person studies might afford. Future research should consider these factors when utilizing the database, as well as whether spaced and unspaced compound words carry similar ratings on the measures collected here (cf. Inhoff et al., 2000; Juhasz, 2005; Kuperman & Bertram, 2013).

In spite of these challenges, however, we find that our participants report a range of experiences with individual constituents, and these experiences vary widely across the three ratings collected here for spaced compound words. In general, these ratings reveal that words with simpler structures (i.e., words with fewer characters, syllables, morphemes, and smaller OLD values) are more frequent and acquired earlier. When these words are encountered in the context of a spaced compound, readers find those compound words to likewise be more familiar and acquired earlier, in addition to contributing to a more transparent meaning for the compound word. Simplicity in a constituent’s structure seems to be especially relevant for second constituents, suggesting that the word order for a compound word may generally reflect a morphological principle that prioritizes salient information by mentioning it earlier, making the second constituent more predictable (cf. Gundel et al., 1993). That is, for an English compound word like *amusement park*, the first constituent *amusement* is longer, contains more syllables, and has more morphemes, than the second constituent. It is also the case that *amusement* is less frequent than *park*, suggesting it is the marked term with a greater information density (Jaeger & Levy, 2006). When paired together as a compound word, then, the front-loading of information allows the reader to form stronger predictions about the second constituent (at least in English).

Indeed, when multiple words are paired together, or frequently appear together in recurring orders, readers not only begin to form expectations as to the word that is likely to follow the first of the pair, but they also likely rely on these expectations to facilitate processing during reading (Diessel, 2007). Through such processing, we consider it likely that commonly co-occurring words develop into established lexical units and that their processing becomes automatized and operationalized over the entire chunk or unit (Zang, 2019). Overall, we intend for the English Spaced Compound Word Database to be a valuable resource for researchers of reading comprehension and mental models of lexical retrieval. We anticipate that it will assist those examining how readers lexically process spaced compound (and other) words, and how such word sequences are processed during natural reading. This, in turn, should directly inform the debate between serial and parallel models of eye movement control during reading. In our view, the database addresses both practical and theoretical gaps in the current research landscape, and we hope that it expands access to a new set of underexplored stimuli for future researchers.

## Data Availability

The materials used in this study and the anonymous data collected for this study are available at https://osf.io/65prn. The file containing the code used to analyze the statistical data is also available in the repository. The study was not preregistered.

## Footnotes

1 As discussed below, the items in the database are sourced from all multi-word entries in Webster’s Dictionary for Students (6th ed.). We have attempted to be as inclusive as possible with respect to the stimuli. For this reason, entries that may or may not carry unique semantic representations for an individual language user are included. We refer to all of the included items as spaced compound words for convenience, as they co-occur enough to be included in at least one authoritative language source.

2 Notably, there also exists a large database of “pseudo-compound” words (Auch et al., 2024). Pseudo-compounds appear to consist of two constituents, but neither is related to the meaning or morphological structure of the entire word. For example, *carpet* is a monomorphemic word that contains two words (*car* and *pet*) that are not related to the morphological structure of the word. While pseudo-compounds are beyond the scope of the present database, there is a growing body of research examining the processing of these items (e.g., Auch et al., 2024; Gagné et al., 2023; Park et al., 2024).

3 In a few cases, participants who did not pass the attention checks were included anyway. In these cases, the participant contacted the experimenter because they were unsure how to respond to the attention checks correctly. Other metrics for these participants (e.g., study duration, average ratings) were within normal range.

4 We retained only the most frequent form of the associated words (or the root word when frequency data were unavailable).

5 Words with four or more constituents were removed. Words with three or more constituents with at least one hyphenation were removed. Words with three constituents that were not hyphenated (e.g., *crossword puzzle*) were considered edge cases and were included (*n* = 34). In these cases, the unspaced constituent (e.g., *crossword*) was treated as a single constituent. These entries are labeled as “Xconst” in the available dataset.

6 The code used for all analyses is included in the OSF repository: https://osf.io/65prn.
